# Altered lymphatic drainage patterns in re-operative sentinel lymph node biopsy for ipsilateral breast tumor recurrence

**DOI:** 10.1186/s13014-019-1367-0

**Published:** 2019-09-02

**Authors:** Ayaka Sato, Takehiko Sakai, Takuji Iwase, Fumiko Kano, Kiyomi Kimura, Akiko Ogiya, Mitsuru Koizumi, Masahiko Tanabe, Rie Horii, Futoshi Akiyama, Takayuki Ueno, Shinji Ohno

**Affiliations:** 1 Department of Breast Surgical Oncology, Breast Oncology Center, The Cancer Institute Hospital of Japanese Foundation for Cancer Research, 3-8-31, Ariake, Koto, Tokyo, 135-8550 Japan; 2 Department of Breast and Endocrine Surgery, The University of Tokyo Hospital, 7-3-1 Hongo, Bunkyo-ku, Tokyo, 113-8655 Japan; 3 Division of Gene regulation, Institute of Advanced Medical Research, Keio University School of Medicine, 35 Shinanomachi, Shinjuku-ku, Tokyo, 160-8582 Japan; 4 Department of Nuclear Medicine, The Cancer Institute Hospital of Japanese Foundation for Cancer Research, 3-8-31, Ariake, Koto, Tokyo, 135-8550 Japan; 5 Department of Pathology, Clinicopathology Center, The Cancer Institute Hospital of Japanese Foundation for Cancer Research, 3-8-31, Ariake, Koto, Tokyo, 135-8550 Japan; 6 Division of Pathology, Clinicopathology Center, The Cancer Institute of Japanese Foundation for Cancer Research, 3-8-31, Ariake, Koto, Tokyo, 135-8550 Japan

**Keywords:** Breast cancer, Lymphoscintigraphy, Aberrant lymphatic drainage, Sentinel lymph node biopsy, Whole breast irradiation, Ipsilateral breast tumor recurrence, Contralateral axilla

## Abstract

**Background:**

This study aimed to evaluate the impact of previous local treatment on lymphatic drainage patterns in ipsilateral breast tumor recurrence (IBTR) based on our data on re-operative sentinel lymph node biopsy (re-SLNB) for IBTR.

**Methods:**

Between April 2005 and December 2016, re-SLNB using lymphoscintigraphy with Tc-99 m phytate was performed in 136 patients with cN0 IBTR. Patients were categorized into two groups: the AX group included 55 patients with previous axillary lymph node dissection; the non-AX group included 69 patients with previous SLNB and 12 patients with no axillary surgery. The whole breast irradiation (RT) after initial surgery had performed in 17 patients in the AX group and 27 patients in the non-AX group.

**Results:**

Lymphatic drainage was visualized in 80% of the AX group and 95% of the non-AX group (*P* < 0.01). The visualization rate of lymphatic drainage was associated with the number of removed lymph nodes in prior surgery. In the non-AX group, lymphatic drainage was visualized in 96% of patients without RT and 93% with RT. Lymphatic drainage was observed at the ipsilateral axilla in 98% of patients without RT and in 64% with RT (*P* < 0.0001). Aberrant drainage was significantly more common in patients with RT than without RT (60% vs. 19%, *P* < 0.001); it was observed mostly to the contralateral axilla (52% vs. 2%, *P* < 0.0001). In the AX group, patients with previous RT showed decreased lymphatic drainage to the ipsilateral axilla compared to those without RT (29% vs. 63%, *P* < 0.05) and increased aberrant drainage to the contralateral axilla (64% vs. 5%, *P* < 0.0001).

**Conclusion:**

Lymphatic drainage patterns altered in re-SLNB in patients with IBTR and previous ALND and RT were associated with alterations in lymphatic drainage patterns.

## Background

While sentinel lymph node biopsy (SLNB) is a well-established procedure for patients with clinically node-negative primary breast cancer [1–4], established guidelines for the management of axillary lymph nodes in patients developing ipsilateral breast tumor recurrence (IBTR) are lacking.

It is known that a sentinel lymph node (SLN) is sometimes observed in extra-axillary regions of IBTR cases because lymphatic drainage patterns are altered by previous treatments, such as axillary surgery and irradiation of the breast [5–8]. Therefore, assessment of the ipsilateral axilla alone may not be sufficient for staging IBTR.

According to a report on SLNB for patients with primary breast cancer, the visualization rate of lymphatic drainage was 97%, and lymphatic drainage was mainly observed to the ipsilateral axilla (96%) [9]. In a small study, aberrant drainage was reported to the internal mammary chain (IMC) (22%), intramammary region (7%), subclavicular region (3%), supraclavicular region (0.5%), and interpectoral region (2%) [9]. In contrast, a meta-analysis of re-operative SLNB (re-SLNB) for IBTR revealed a success rate of 71% for lymphatic mapping, markedly lower than that of SLNB in patients with primary breast cancer [10]. Aberrant drainage was observed in 43% of these patients, a much higher frequency than in patients with primary breast cancer. Although it is well known that previous axillary lymph node dissection (ALND) decreases the identification rate of SLNs for IBTR and increases aberrant drainage [5–7, 11], the impact of previous radiotherapy (RT) on re-SLNB remains largely unknown.

Because re-SLNB may provide useful information in determining adjuvant treatment for IBTR, we have performed re-SLNB for IBTR, using radioisotope techniques and preoperative lymphoscintigraphy to stage IBTR. The aim of this retrospective study was to evaluate lymphatic drainage patterns in re-operative SLNs (re-SLNs) in association with prior local therapy in patients with IBTR.

## Methods

The institutional clinical database was used to identify patients who developed IBTR and underwent re-SLNB between April 2005 and December 2016. The ethical review committee of the institute approved this study protocol (No.2018–1222). Informed consent was obtained from all individual participants included in the study. Patients who underwent re-SLNB without preoperative lymphoscintigraphy, those with synchronous or metachronous bilateral breast cancer, and those lacking detailed information on previous surgeries were excluded. None of the patients had clinically metastatic lymph nodes, as examined by preoperative ultrasound. Fine needle aspiration cytology was performed if node metastasis was suspected by ultrasound.

The day before surgery, total 55.5 MBq (1.5 mCi) Tc-99 m phytate was injected at two intradermal sites at the tumor and at two peritumoral sites. Static images were obtained 1 h after the injection from 3 projections (anterior, 30 degrees anterior-oblique, and 60 degrees anterior-oblique views). Hot spots on the lymphoscintigram were regarded as re-SLNs. SPECT/CT was also performed in a subset of patients.

Patients were categorized into two groups according to their previous axillary surgeries: the non-AX group included patients with SLNB and no previous axillary surgery, and the AX group included those with ALND. Patients were further categorized based on the use of previous adjuvant RT.

A chi-squared test was applied to evaluate differences in lymphatic drainage patterns between the AX group and the non-AX group and between the RT group and the no RT group. The Mann-Whitney test was used to compare the number of removed nodes between different groups. The log-rank test was used to compare the disease-free interval (DFI), the interval from primary surgery to the diagnosis of IBTR, between the patients whose lymphatic drainage was visualized on lymphoscintigraphy and these whose lymphatic drainage was not visualized. GraphPad Prism v.5.04 (GraphPad Software, San Diego, CA, USA) was used for statistical analysis. A two-sided *P* value of < 0.05 was considered statistically significant.

## Results

### Characteristics of patients

Between April 2005 and December 2016, 277 patients were identified who developed IBTR and underwent re-SLNB. Of the 277 patients, 141 were excluded according to the exclusion criteria, with 136 patients remaining in the analysis. The characteristics of the 136 patients are shown in Table 1. Median age at IBTR was 55 years. The median DFI was 67 months. Median follow-up period from the day of surgery for IBTR was 141 months.

**Table 1:** Characteristics of patients with primary breast cancer and ipsilateral breast tumor recurrence

	*N* = 136
Ipsilateral breast tumor recurrence	
Median age (years old)	55 (30–79)
Median disease-free interval (months)^a^	67 (10–233)
Clinical T stage	
Tis	30
T1	90
T2	15
T3	1
Median follow-up period (months)	141 (32–315)
At primary surgery	
Median age (years old)	47 (22–76)
Clinical T stage of primary breast cancer	
Tis	23
T1	72
T2	34
T3	5
unknown	2
Surgery for axilla	
no axillary surgery	12
SLNB^b^	69
ALND^c^	55
Adjuvant radiotherapy (RT)	
Without RT	92
With RT	44

^a^disease-free interval: the interval from primary surgery until the day ipsilateral breast tumor recurrence was diagnosed

^b^ SLNB: sentinel lymph node biopsy

^c^ ALND: axillary lymph node dissection

Median age at primary surgery was 47 years. All patients had undergone breast-conserving surgery for their primary breast cancer. Sixty-nine and 55 patients had undergone SLNB and ALND, respectively, and 12 patients had no previous axillary surgery. Median number of lymph nodes removed at the primary surgery was 2 (1–6) in patients who had undergone SLNB and 20 (8–34) in patients who had undergone ALND. RT after breast-conserving surgery was performed in 44 patients. The whole breast irradiation dose was 42.5–50Gy with or without a boost dose of 10–16Gy in tumor beds. RT was not performed when surgical margins were entirely free of cancer, as confirmed by a precise pathological examination according to our institutional treatment protocol [12].

### Visualization of lymphatic drainage patterns on lymphoscintigraphy

**Fig. 1:**
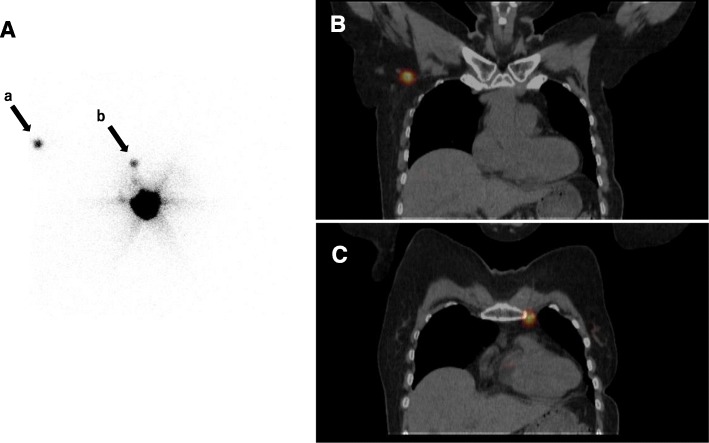
Lymphoscintigraphy and SPECT/CT images of aberrant lymphatic drainages. (**A**) Lymphatic drainages were visualized at the contralateral axilla (arrow a) and the ipsilateral internal mammary chain (IMC) (arrow b) in a case with left IBTR. SPECT/CT revealed hot spots at the right axillary region (**B**) and the left IMC (**C**)

Preoperative lymphoscintigraphy identified at least one SLN, defined as re-SLN, in 121 (89%) of the 136 patients (Table 2). Lymphatic drainage was visualized at the ipsilateral axilla in 74%. Some patients showed multiple patterns of lymphatic drainage. Aberrant drainage was visualized in five regions: IMC, supraclavicular, intramammary, contralateral axilla, and contralateral IMC (Table 2). A representative case of aberrant drainage is shown in Fig. 1. DFI was shorter in patients whose lymphatic drainage was visualized on lymphoscintigraphy compared with patients whose lymphatic drainage was not visualized (60 vs. 129 months, *P* < 0.05). No difference in visualization rate was observed according to hormone receptor status and HER2 status for both primary cancer and IBTR (Table 3).

**Table 2:** Lymphatic drainage patterns in all 136 patients with IBTR^a^

	*N* = 136
Visualization on lymphoscintigraphy	
Yes	121 (89.0%)
No	15 (11.0%)
Lymphatic drainage patterns	
Ipsilateral axilla	90 (74.4%)
Aberrant drainage	57 (47.1%)
Internal mammary chain (IMC)	36 (29.8%)
Contralateral axilla	25 (20.7%)
Intramammary	9 (7.4%)
Supraclavicular	2 (1.7%)
Contralateral IMC	1 (0.8%)

Lymphatic drainage was visualized in 121 of 136 patients. Lymphatic drainage to ipsilateral axilla was visualized in 74% of patients. Aberrant drainage was visualized in five regions: internal mammary chain (IMC), supraclavicular, intramammary, contralateral axilla, and contralateral IMC

^a^*IBTR* ipsilateral breast tumor recurrence

**Table 3:** Visualization on lymphoscintigraphy according to hormone receptor and HER2 status of primary tumor and IBTR

	Visualization on lymphoscintigraphy (*N* = 136)		
	Yes	No	*P*
a. Primary breast cancer			
ER^a^			
Positive	54	3	ns^d^
Negative	25	6	
DCIS^b^	26	2	
unknown	16	4	
PgR^c^			
Positive	46	5	ns
Negative	32	4	
DCIS	26	2	
Unknown	17	4	
HER2			
3+	8	0	ns
2+	5	0	
1+, 0	40	6	
DCIS	26	2	
Unknown	42	7	
b. Ipsilateral breast tumor recurrence			
ER			
Positive	64	10	ns
Negative	26	2	
DCIS	29	2	
Unknown	2	1	
PgR			
Positive	46	7	ns
Negative	44	5	
DCIS	29	2	
unknown	2	1	
HER2			
3+	10	0	ns
2+	0	0	
1+, 0	78	12	
DCIS	29	2	
Unknown	4	1	

The visualization on lymphoscintigraphy was not associated with hormone receptor status and HER2 status of both primary cancer and IBTR

^a^ER: estrogen receptor

^b^DCIS: ductal carcinoma in situ

^c^PgR: progesterone receptor

^d^ns: no significant difference

### Impact of axillary surgery on lymphatic drainage patterns

Lymphatic drainage patterns were compared according to previous axillary surgeries. The visualization rate of lymphatic drainage was higher in the non-AX group (95%) than in the AX group (80%) (*P* < 0.01) (Table 4). The visualization rate was associated with the number of lymph nodes which had been removed in the prior surgery (Table 5). The median number of removed lymph nodes in the prior surgery was fewer in patients whose lymphatic drainage was visualized on lymphoscintigraphy compared with patients whose lymphatic drainage was not visualized (8 vs. 16 nodes, *P* < 0.05). Lymphatic drainage was visualized in all 12 patients who had not undergone previous axillary surgery (Table 5). The visualization rate was markedly low (71%) in patients in whom 20 or more lymph nodes had been removed in prior surgery (Table 5).

**Table 4:** Lymphatic drainage patterns according to previous axillary surgery

	Previous axillary surgery		
	non-AX group^a^	AX group^b^	
	*N* = 81	*N* = 55	*P* value
Visualization on lymphoscintigraphy			
Yes	77 (95.1%)	44 (80.0%)	< 0.01
No	4 (4.9%)	11 (20.0%)	
Lymphatic drainage patterns			
Ipsilateral axilla	67 (87.0%)	23 (52.2%)	< 0.001
Aberrant drainage	25 (32.5%)	33 (75.0%)	< 0.0001
Internal mammary chain (IMC)	12 (15.6%)	24 (54.5%)	< 0.001
Contralateral axilla	14 (18.2%)	11 (25.0%)	ns^c^
Intramammary	6 (7.8%)	3 (6.8%)	ns
Supraclavicular	0 (0%)	2 (4.5%)	ns
Contralateral IMC	1 (1.3%)	0 (0%)	ns

Lymphatic drainage patterns were compared among the 136 patients according to previous axillary surgeries. The visualization rate of re-SLNs in the non-AX group was higher than in the AX group. Aberrant drainages were visualized more frequently in the AX group than in the non-AX group. Whereas re-SLNs were visualized at the ipsilateral axilla in about 87% of the non-AX group, drainage to the ipsilateral axilla was significantly decreased and an alternative aberrant drainage pattern to the internal mammary chain was significantly increased in the AX group

^a^ non-AX group: patients with previous SLNB and no previous axillary surgery

^b^ AX group: patients with previous axillary lymph node dissection

^c^ ns: no significant difference

**Table 5:** The visualization rate on lymphoscintigraphy according to the number of lymph nodes removed in prior surgery

Number of lymph nodes removed in prior surgery	The visualization rate of re-SLNs^a^
Non-AX group^b^	
0	100% (12/12)
1–2	94.3% (33/35)
3–6	93.8% (30/32)
AX group^c^	
8–19	91.7% (22/24)
20 or greater	71.4% (20/28)

^a^re-SLNs: re-operative sentinel lymph nodes

^b^non-AX group: patients with previous SLNB and no previous axillary surgery

^c^AX group: patients with previous axillary lymph node dissection

Number of lymph nodes which had been removed in prior surgery was unknown in two patients of non-AX group and three patients of AX group

The visualization rate was associated with the number of lymph nodes which had been removed in the prior surgery. Lymphatic drainage was visualized in all patients who had not undergone previous axillary surgery. The visualization rate was markedly low in patients in whom 20 or more lymph nodes had been removed in prior surgery

Lymphatic drainage was visualized at the ipsilateral axilla in 87% of patients in the non-AX group and in 52% in the AX group (*P* < 0.001, Table 4). Aberrant drainage was visualized significantly more frequently in the AX (75%) than in the non-AX (33%) group (*P* < 0.0001, Table 4). Lymphatic drainage was visualized at the IMC in 16% of the non-AX group and 55% of the AX group (*P* < 0.001, Table 4). Although axillary dissection had been performed in the AX group, re-SLNs were visualized in the ipsilateral axilla in 52% of patients: at level I and II of the axillae in seven patients, in the Rotter space in ten patients, and at level III of the axilla in three patients. In three patients in the non-AX group, re-SLNs that were visualized by lymphoscintigraphy failed to be identified during surgery because of the poor responses of the gamma-ray detection probe.

### Impact of adjuvant radiotherapy on lymphatic drainage patterns

In the non-AX group, lymphatic drainage was visualized in 96% of patients without RT and 93% of those with RT (Table 6a). Re-SLNs were visualized at the ipsilateral axilla in 98% of the patients without RT and in 64% of those with RT (*P* < 0.0001, Table 6a). Aberrant drainage was significantly more frequent in patients with RT (60%) than in those without RT (19%) (*P* < 0.001). Notably, more than half (52%) of patients with RT showed aberrant drainage to the contralateral axilla, whereas only 2% of those without RT did (*P* < 0.0001). No re-SLNs were visualized in the supraclavicular region in the non-AX group.

In the AX group, lymphatic drainage was visualized in 82% of patients with RT and 79% of those without RT (Table 6b). Aberrant drainage to the contralateral axilla was observed in nearly two-thirds (64%) of patients with RT but in 5% of those without RT (*P* < 0.0001, Table 6b).

**Table 6:** Lymphatic drainage patterns according to previous radiotherapy

a) non-AX group^a^			
	Without RT^c^	With RT	
	*N* = 54	*N* = 27	*P* value
Visualization on lymphoscintigraphy			
Yes	52 (96.3%)	25 (92.6%)	ns^d^
No	2 (3.7%)	2 (7.4%)	
Lymphatic drainage patterns			
Ipsilateral axilla	51 (98.1%)	16 (64.0%)	< 0.0001
Aberrant drainage	10 (19.2%)	15 (60.0%)	< 0.001
Internal mammary chain (IMC)	7 (13.5%)	5 (20.0%)	ns
Contralateral axilla	1 (1.9%)	13 (52.0%)	< 0.0001
Intramammary	4 (7.7%)	2 (8.0%)	ns
Supraclavicular	0 (0%)	0 (0%)	ns
Contralateral IMC	0 (0%)	1 (4.0%)	ns
b) AX group^b^			
	Without RT	With RT	
	*N* = 38	*N* = 17	*P* value
Visualization on lymphoscintigraphy			
Yes	30 (78.9%)	14 (82.4%)	ns
No	8 (21.1%)	3 (17.6%)	
Lymphatic drainage patterns			
Ipsilateral axilla	19 (63.3%)	4 (28.6%)	< 0.05
Aberrant drainage	20 (66.7%)	13 (92.9%)	ns
Internal mammary chain (IMC)	17 (56.7%)	7 (50%)	ns
Contralateral axilla	2 (5.3%)	9 (64.3%)	< 0.0001
Intramammary	3 (10.0%)	0 (0%)	ns
Supraclavicular	1 (3.3%)	1 (7.1%)	ns
Contralateral IMC	0 (0%)	0 (0%)	

Lymphatic drainage patterns were compared among the patients according to the presence or absence of previous radiotherapy in the non-AX group and the AX group. RT had little impact on the visualization rate of re-SLNs in both groups (Table 6a, b). In the non-AX group, lymphatic drainage to ipsilateral axilla was significantly decreased and aberrant drainage to contralateral axilla was significantly increased in patients with RT (Table 6a). In the AX group, re-SLNs were visualized at contralateral axilla more commonly in patients with RT (Table 6b)

^a^non-AX group: patients with previous SLNB and no previous axillary surgery

^b^AX group: patients with previous axillary lymph node dissection

^c^RT: previous radiotherapy after breast-conserving surgery

^d^ns: no significant difference

## Discussion

The present study revealed that the previous local treatment, not only axillary surgery but also RT, had impact on lymphatic drainage patterns in patients with IBTR. First, we confirmed the impact of previous axillary surgery on lymphatic drainage patterns. Next, we demonstrated that previous RT resulted in aberrant lymphatic drainage in 60% of patients in the non-AX group and in 93% in the AX group.

We found that the visualization rate of re-SLN was almost the same regardless of previous RT while it was reduced by axillary surgery (Tables 4, 6). In addition, RT reduced the ipsilateral axillary drainage from 98 to 64% and increased the aberrant drainage, especially to the contralateral axilla both in the AX group and in the non-AX groups. There were a few studies that examined the impact of RT on lymphatic drainage patterns and showed that RT had no effect on the identification rate of re-SLNs, in concordance with our study [5, 13]. Interestingly, in a study with 22 patients with breast cancer who had previously undergone mantle field radiation for Hodgkin’s lymphoma, the detection rate of SLNs was 86%, which was less than that in previously untreated patients with breast cancer (97%) [14]. The visualization rate of SLNs at the ipsilateral axilla was lower than that in patients with previously untreated breast cancer (86% vs. 92%), and SLNs were more often found outside the axilla (41% vs. 33%), especially at the IMC (32% vs. 21%) [14]. The discrepancy between our results and those of the patients receiving mantle field radiation could be attributed to the difference in the irradiated fields and doses. In the study of mantle field radiation, the lymphatic regions in the neck, bilateral axilla, and mediastinum received a radiation dose of 36–40 Gy [14]. The medial and upper outer quadrants of the breast were also exposed to radiation. In contrast, the whole breast irradiation dose was 42.5–50Gy in the present study, and some patients were also given a boost dose of 10–16Gy in tumor beds. Considering that lymphatic drainage had been damaged by irradiation and aberrant lymphatic drainage did not involve the irradiated area, it was understandable that lymphatic drainage to the IMC increased after patients received mantle field irradiation and that lymphatic drainage to the contralateral axilla increased in those with previous whole breast irradiation.

According to our results, in patients with IBTR with previous SLNB and non-RT, lymphatic drainage remains mostly in the ipsilateral axilla, and these patients will be suitable for re-SLNB. On the other hand, re-SLNs were visualized at the contralateral axilla in more than 20% of the whole population (Table 2). Although there are some reports showing the feasibility of the re-SLNB procedure [7, 15–18], the clinical significance of re-SLNB has not yet been confirmed. In addition, it is further complicated in cases with aberrant SLNs. Metastasis to the contralateral axilla is regarded as distant metastasis in primary breast cancer [19], but in patients with IBTR, the contralateral axilla may be considered a regional lymph node if re-SLNs are identified in the contralateral axilla [20]. Although there are no guidelines for management of contralateral SLN and positive contralateral SLN, pathological results in contralateral SLN may be useful in deciding adjuvant treatment for IBTR [21]. Further studies to clarify the clinical significance of aberrant SLNs are required.

The major limitation of this study is the retrospective design with a small number of patients. In particular, the number of patients who had received previous RT was small and not enough to make any conclusion on the impact of RT on lymphatic drainage patterns. In addition, this study focuses only on lymphatic drainage patterns and does not examine the success rate of SLNB or pathological results of re-SLNs. Thus, the clinical significance of re-SLN examination is not clear. Another limitation is the lack of information on patient outcomes. It is important to examine the clinical outcomes of patients in association with treatment, including surgical procedures to determine the optimal treatment strategy. This is especially important in patients with aberrant lymphatic drainage.

## Conclusion

In conclusion, lymphatic drainage patterns altered in re-SLNB in patients with IBTR and previous ALND and RT were associated with alterations in lymphatic drainage patterns. More studies focusing on the clinical usefulness of re-SLNB, including outcomes after re-SLNB, are warranted.

## Data Availability

All data generated or analyzed during this study are included in this published article.

## Abbreviations

ALND: Axillary lymph node dissection
DFI: Disease-free interval
IBTR: Ipsilateral breast tumor recurrence
IMC: The internal mammary chain
re-SLNB: Re-operative sentinel lymph node biopsy
re-SLNs: Re-operative sentinel lymph nodes
RT: Radiotherapy
SLN: Sentinel lymph node
SLNB: Sentinel lymph node biopsy
